# Cerebral rScO2 Measured by Near-Infrared Spectroscopy (NIRS) During Therapeutic Hypothermia in Neonates with Hypoxic-Ischemic Encephalopathy: A Systematic Review

**DOI:** 10.34763/jmotherandchild.20242801.d-24-00010

**Published:** 2024-04-19

**Authors:** Sergio Agudelo-Pérez, Gloria Troncoso, Alejandra Roa, Ana Gabriela Ariza, Georgina Doumat, Natalia M. Reinoso, Daniel Botero-Rosas

**Affiliations:** PhD. Health Sciences. Pediatric Neonatologist. Associate professor, Deparment of Pediatrics, School of Medicine, Universidad de La Sabana. Campus Puente del Común, Km 7, Autopista Norte de Bogotá, Chía, Cundinamarca, Colombia; Pediatric Neonatologist. Head Neonatal Unit. Fundación Cardioinfantil - Instituto de Cardiología. Bogotá, Colombia; Medical student. School of Medicine, Universidad de La Sabana. Chía, Cundinamarca, Colombia; Biomedical engineering, MSc. Biomedical engineering. Associate professor. Department of Bioscience. School of Medicine. Universidad de La Sabana. Chía, Cundinamarca, Colombia

**Keywords:** Newborn, Hypoxic-ischemic encephalopathy, Asphyxia, Hypothermia Therapeutic, Spectroscopy Near-Infrared

## Abstract

**Introduction:**

Perinatal asphyxia, a leading cause of neonatal mortality and neurological sequelae, necessitates early detection of pathophysiological neurologic changes during hypoxic-ischaemic encephalopathy (HIE). This study aimed to review published data on rScO2 monitoring during hypothermia treatment in neonates with perinatal asphyxia to predict short- and long-term neurological injury.

**Methods:**

A systematic review was performed using the Preferred Reporting Items for Systematic Reviews and Meta-Analysis (PRISMA) guidelines. Study identification was performed through a search between November and December 2021 in the electronic databases PubMed, Embase, Lilacs, Scopus, Web of Science, and Cochrane Central Register of Controlled Trials (CENTRAL). The main outcome was short-term (Changes in brain magnetic resonating imaging) and long-term (In neurodevelopment) neurological injury. The study protocol was registered in PROSPERO (International Prospective Register of Systematic Reviews) with CRD42023395438.

**Results:**

380 articles were collected from databases in the initial search. Finally, 15 articles were selected for extraction and analysis of the information. An increase in rScO2 measured by NIRS (Near-infrared spectroscopy) at different moments of treatment predicts neurological injury. However, there exists a wide variability in the methods and outcomes of the studies.

**Conclusion:**

High rScO2 values were found to predict negative outcomes, with substantial discord among studies. NIRS is proposed as a real-time bedside tool for predicting brain injury in neonates with moderate to severe HIE.

## Introduction

Perinatal asphyxia is one of the main causes of neonatal mortality and medium- and long-term neurological sequelae in the paediatric age group [1]. For low- and middle-income countries and clinical settings, it is defined as failure to initiate or maintain breathing at birth [2]. The current treatment for neonates with moderate to severe asphyxia is therapeutic hypothermia initiated in the first six hours of life [3] to reduce mortality, morbidity, and the severity of neurological sequelae [4]. However, medium- and long-term sequelae remain high [5].

Therefore, the early detection of pathophysiological alterations at the neurological level during hypoxic-ischaemic encephalopathy before and during treatment that can predict brain damage would allow therapeutic interventions to be instituted in the early stages of the disease, establish follow-up before discharge from the unit in high-risk neurological programmes, and deepen the knowledge of the mechanisms of neurological injury in the phases of asphyxia. In this area, neuromonitoring during the 72 hours of cooling and rewarming has been established as an option to recognise cerebral hypoperfusion, encephalopathy, abnormal electrical activity, and, in general, physiological, clinical, and/or biochemical variables that serve as biomarkers for relevant identification [6].

Currently, neuromonitoring is centred on electroencephalography (EEG), amplitude-integrated electroencephalography (aEEG), electroencephalogram, and video telemetry [7]. These electrophysiological tests during cooling have shown usefulness in defining prognosis and the presence of brain injury in the first six hours of treatment [6]. However, abnormal tracing after six hours, especially in the first 24 to 36 hours, has a low predictive value [8]. On the other hand, the presence of abnormalities in brain magnetic resonance imaging (MRI) in the first week of life has a high predictive value for neurodevelopmental alteration and neurological sequelae in the medium and long term. However, its findings are late and changeable in the evolution of perinatal asphyxia, so when the alteration is found in the images, there is already an established neurological injury [9].

Near-infrared spectroscopy (NIRS) is a non-invasive, portable, and reproducible bedside monitoring technique within the neonatal intensive care unit without potential harm to the neonate [14]. It provides continuous, early, and timely information on regional cerebral haemoglobin oxygen saturation, cerebral blood volume, and cerebral oxygen supply/metabolism ratio, making it a potential technique for neuromonitoring in neonates, especially in brain tissue hypoxia and associated injuries [15]. Continuous monitoring of cerebral regional oxygen saturation (rScO2) would allow timely diagnosis and may have the ability to predict early brain injury during the early stages of neonatal asphyxia. In addition, together with other clinical monitoring variables, it could identify pathophysiological alterations in cerebral blood flow [9]. Studies in animal models show the potential role of NIRS in the timely detection of brain injury [9]. Likewise, research in neonates is promising for the association between changes in and values of rScO2 during hypothermia and the future presence of neurological injury [10].

Integrating different tests when monitoring brain functions, from the electrophysiological perspective, metabolism, and oxygenation, would allow the optimal and timely identification of the neonate at risk of injuries and neurological alterations [9]. Additionally, predicting neurological outcomes among neonates with hypoxic-ischaemic encephalopathy (HIE) has become a critical and ongoing challenge. This study aimed to conduct a systematic literature review of published data on rScO2 monitoring, measured by NIRS, during hypothermia treatment in neonates with perinatal asphyxia to predict short- and long-term neurological injury. The secondary objectives were to characterise the quality of the studies entered and to explore heterogeneity. This study helps to characterise the current status of rScO2 by NIRS in neurological injury in neonates with asphyxia and may provide a basis for future research design.

## Methods

The study protocol was registered in PROSPERO (International Prospective Register of Systematic Reviews) with CRD code 42023395438.

### Identification of studies

A systematic review was performed using the PRISMA guidelines [11] to identify, screen, and include studies. Study identification was performed through a search between November and December 2021 in the electronic databases PubMed, Embase, Lilacs, Scopus, Web of Science, and Cochrane Central Register of Controlled Trials (CENTRAL). The terms used were newborn, infant, hypoxic-ischaemic encephalopathy, asphyxia, hypothermia therapeutic, and spectroscopy near-infrared. The following search strategy was used for PubMed and matched to the other databases: ((-newborn, infant) AND (((hypoxic-ischaemic encephalopathy) OR (asphyxia)) OR (hypothermia therapeutic))) AND (spectroscopy near-infrared). No restriction was made by language and year. We planned to include analytical observational studies and/or clinical trials using rScO2 neuromonitoring by NIRS during hypothermia treatment to predict short- and long-term neurological injuries. Likewise, to report the sensitivity, specificity, and/or predictive values for the discrimination of neonates with neurological injury following therapeutic hypothermia.

### Inclusion criteria

1. Newborns born at term (gestational age greater than or equal to 37 weeks at birth) and/or near term (greater than or equal to 35 weeks) with a diagnosis of moderate to severe asphyxia and/or moderate to severe hypoxic-ischaemic encephalopathy who had received treatment with therapeutic hypothermia in the first six hours of life.
2. Neuromonitoring of rScO2 by NIRS during the duration of treatment with hypothermia and/or rewarming.
3. Analytical observational studies and/or clinical trials evaluating the discriminative and/or predictive capacity of rScO2 for the identification of brain injury in the short, medium, and long-term.

### Exclusion criteria

1. Secondary articles.
2. Case reports.
3. Abstracts of articles, posters, and/or conference presentations.

### Outcome

The main short-term outcome was defined as neurological injury before discharge from the neonatal unit: a) brain MRI alteration compatible with perinatal asphyxia sequelae; b) EEG alteration compatible with perinatal asphyxia sequelae; c) presence of seizures before discharge and/or alteration of the neurological examination. On the other hand, the medium- and short-term outcomes were defined as neurodevelopmental impairment and/or cerebral palsy in the first 36 months of follow-up.

### Screening and inclusion of studies

The search and selection of studies were performed independently by three investigators (AAS, DRG, and GDC). Studies identified in the initial search were screened by title and abstract in the Rayyan® web tool [12], where duplicate records due to overlap between the databases consulted were determined and eliminated. The initial results were compared, and discrepancies were resolved in consensus with two additional investigators (SAP and JB). Subsequently, relevant articles were obtained for full-text reading by the authors independently to define their final entry into the systematic review, and discrepancies were resolved by consensus.

The extraction of information was performed using an Excel® instrument, including bibliographic data (author, year of publication) and relevant data on the type of study (methodology), methodology for NIRS measurement, population (term and/or near-term neonates), type of neurological injury present as an outcome, the NIRS cut-off value used for prediction of neurological damage, and the NIRS cut-off value used for prediction of neurological damage.

### Evaluation of the methodological quality of the studies

The assessment of the risk of bias in the observational studies was conducted with the STROBE (Strengthening the Reporting of Observational Studies in Epidemiology) checklist. On the other hand, for clinical trials, the assessment of bias was planned with the “risk of bias” tool of the Cochrane Collaboration.

### Synthesis of information

Data synthesis was planned according to the NIRS measurement method: technology used (INVOS 5100C, INVOS 4100), sensor position (frontal, frontoparietal, or parietal), measurement time (hypothermia, rewarming), and categorised by defined short- and long-term outcomes (MRI, neurodevelopment).

A quantitative synthesis of the data, or meta-analysis, could not be performed for several reasons, but principally because of the heterogeneity in the time of measurement, the defined outcomes and their diagnostic criteria, the methodology used for rScO2 monitoring, the time of NIRS collection, and the cut-off values to define neurological injury.

## Results

**Figure 1.**
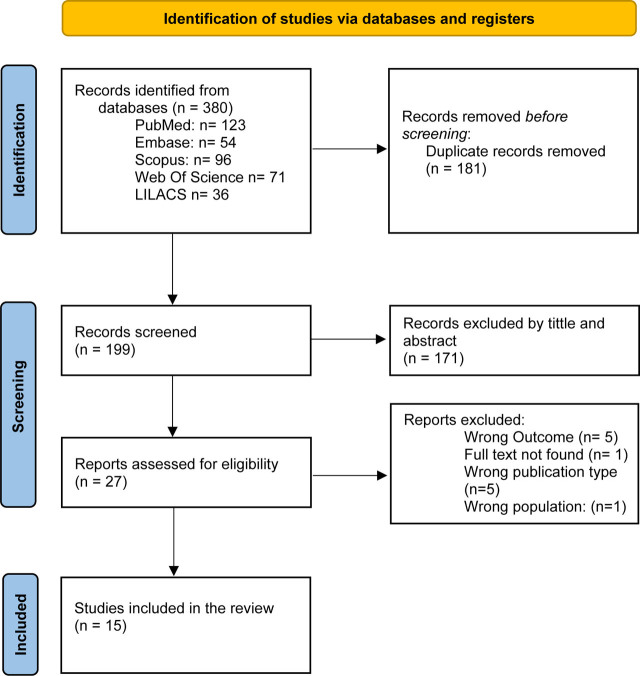
Flowchart of the study.

n = 380 articles were collected from databases in the initial search, of which 181 were eliminated as duplicates when entered into the web tool Rayyan®, resulting in a total of 199 articles being discriminated by title and abstract. From this process, 27 articles met the inclusion criteria for full-text reading, and finally, 15 articles were selected for extraction and analysis of the information. Among the reasons for discarding the remaining ones were incorrect results (n = 5), full text not found (n = 1), types of publication and/or different types of study (n = 5), and incorrect population (n = 1). See Figure 1.

The methodology of the studies was 100% analytical observational cohort studies, 67% prospective, and 33% retrospective. All met the sampling power target set by the authors. The total number of neonates studied was n = 362, including term and near-term neonates. In general, NIRS measurements were performed in frontal, parietal, or frontoparietal locations, of which seven were bilateral and eight unilateral, three parietal, four frontoparietal, and eight frontal, the latter being the most common location.

The neurological outcomes studied included short-term (n = 6) and long-term (n = 5), and n = 4 included both short-term and long-term outcomes. For the identification of short-term outcomes, brain MRI was used in the first days of life, mostly in the first week, in nine studies; amplitude-integrated electroencephalogram (aEEG) in two studies, and only one study evaluated short-term neurodevelopment using the Thompson scale [13]. On the other hand, for the identification of long-term outcomes, neurodevelopment was assessed using BSID III scales (Bailey Scales of Infant Development Version 3) in four studies and GMDS (Griffiths Mental Development Scales) in four studies, while one study implemented Capute and Mullen scales (Table 1).

**Table 1.** Characteristics of included studies.

**BIBLIOGRAPHIC DATA**	**Population**	**Type of study**	**Neurological outcome**	**NIRS measurement**	**Cut-off value**	**Outcome**
Niezen, C et al. 2018	Term and near-term newborns n = 39	Retrospective cohort	SHORT TERM Brain MRI: VanRooij score aEEG: Epileptic activity LONG TERM: Neurodevelopment: BSID-III at 30 months	Left or right frontoparietal	rScO2 >90% at 48 and 72 hrs and in rewarming	rScO2 has no predictive value in the initial phase of HT treatment. After 48 hours its predictive value increases. After rewarming, rScO2 is an even better predictor of outcome than aEEG.
Oliveira Pereira C et al. 2021	Term newborns n=28	Retrospective cohort	LONG TERM: Neurodevelopment: GMDS-Vineland at 18–36 months	Left or right Frontal	rScO2 >82% at 48 hrs and in rewarming	Comparison of rScO2 between the different neurodevelopmental groups revealed statistically significant differences at 48 hours of life between the moderately impaired and severely impaired groups (p = 0.019), and the severely disabled and normal neurodevelopmental groups (p = 0.013).
Szakmar E et al. 2021	Term and near-term newborns n = 49	Retrospective cohort	SHORT TERM Brain MRI: with and without injury	frontoparietal	rScO2>84% in rewarming	The rScO2 value during hypothermia was not statistically significant. The rScO2 during rewarming was higher in infants with brain damage.
Ancora G et al. 2013	Term newborns n=16	Retrospective cohort	LONG TERM: Neurodevelopment: GMDS global quotient <88.7	Bifrontal TOI at 6,12, 24 hrs	-	TOI (Tissue Oxygenation Index) values at 12 hours were significantly higher in infants with adverse events (n-4) vs. those without adverse outcomes (n-8) (79.7 +/− 9.4% vs. 67.1+/− 7.9 p=0.034).
Goeral K et al. 2017	Term and near-term newborns n = 32	Retrospective cohort	SHORT TERM Brain MRI: Gray matter and basal ganglia injury	frontoparietal	-	Values of rScO2 and cFTOE were not significantly different between the two groups (normal and abnormal MRI). A trend of decreased rScO2 and increased cFTOE was observed in patients with normal MRI.
Arriaga-Redondo M et al. 2019	Term and near-term newborns n = 23	Retrospective cohort	SHORT TERM: Brain MRI: Rutherford score LONG TERM: Neurodevelopmental BSID-III at 36 months WISC / GMFCS	left Frontal	rScO2 >90%	rScO2 values >90% and lack of variability in infants with IHD during cooling provide useful information on the severity of neurological status.
Toet M et al. 2006	Term newborns n=18	Retrospective cohort	LONG TERM: Neurodevelopmental GMDS 3, 9, 18, 36 months and 5 years.	Left Parietal FTOE 24 hrs	rScO2 >70% at 24 hrs	rScO2 values increased to supranormal values after 24 h in infants with an adverse outcome. At 24 hr, FTOE values of infants with an adverse outcome were significantly lower compared to those with a favorable outcome.
Wintermark P et al. 2014	Term newborns n=7	Retrospective cohort	SHORT TERM Brain MRI: Gray matter and basal ganglia injury	Bifrontal	-	In the group with adverse outcomes, rScO2 values were significantly higher at 24 hr of life.
Lemmers P et al. 2013	Term and near-term newborns n = 39	Retrospective cohort	SHORT TERM: MRI: basal ganglia lesion, thalamus. LONG TERM Neurodevelopmental GMDS <85 at 18 months	Bilateral frontoparietal FTOEI	-	The mean cFTOE value reflected the rScO2 patterns of both groups and became very low after 24 h of age in the adverse outcome group.
Shellhaas R et al. 2013	Term and near-term newborns n = 21	Retrospective cohort	SHORT TERM: Brain MRI: Low scores no lesion, high lesion more extensive lesion. Neurodevelopmental: Thompson >10 (Before hypothermia and after rewarming).	Biparietal Thigh (systemic)	-	Absolute values and variability of rScO2 was independent of short-term outcome. Systemic rSO2 variability was the best single predictor of short-term outcome scores.
Peng S et al. 2015	Term and near-term newborns n = 18	Retrospective cohort	SHORT TERM: Brain MRI: With/without injury Autopsy: With/without injury	Bifrontal	rScO2 >75.5% in first 10 hours of hypothermia	The rScO2 was higher in asphyxiated neonates who developed subsequent brain injury. This difference was especially prominent during the first 10 hours of hypothermia treatment.
Jain S et al. 2017	Term and near-term newborns n = 21	Retrospective cohort	SHORT TERM: Brain MRI: None/mild, moderate, severe. LONG TERM: Neurodevelopmental: BSID-III 18–24 months	Medial frontal	rScO2>80% at 30 hours of life	The rScO2 increased more rapidly in infants with greater lesion seen on MRI. On average, rScO2increased by 0.20 % per hour when MRI scores 0 or 1, by 0.48 % per hour scores of 2, and by 0.68 % per hour scores of 3. Higher rScO2 beyond 24 h correlated with higher odds of worse BSID scores.
Mitra S et al. 2020	Term newborns n=14	Retrospective cohort	SHORT TERM: aEEG: Mild vs. moderate/severe	Medial frontal	-	The relationship between brain metabolism and oxygenation measured during rewarming after TH in a group of infants with HIE strengthened with increasing degree of brain injury.
Burton V et al. 2015	Term and near-term newborns n = 19	Retrospective cohort	LONG TERM: Neurodevelopmental 2 years, Capute Scale, Mullen.	Bifrontal	-	mean rScO2 in any period (hypothermia, rewarming or normothermia) was not associated with future impairment or Mullen score.
Shellhaas R et al. 2015	Term newborns n=18	Retrospective cohort	LONG TERM: Neurodevelopment at 18 months: BSID III <85	Biparietal	-	There was no relationship between rScO2 and outcome (p>0.05 at all-time points

**Figure 2.**
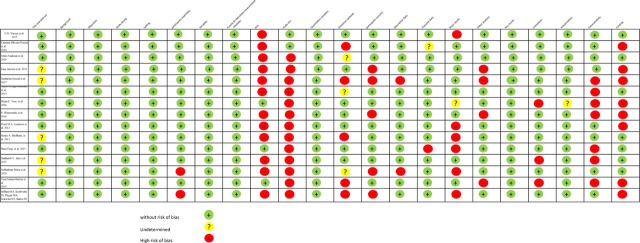
Quality of the selected articles, using STROBE: cohort studies.

Overall, the quality of the studies was adequate, with an average of 78% of the STROBE list for cohort studies (Figure 2). Twenty-two percent of the remaining articles are at high risk of bias, mainly due to the omission of study biases and the failure to describe sample sizes.

### Short-term outcomes

Brain injury by MRI was the main outcome defined in the short term. It was performed on average between the fourth and seventh days of postnatal life. Most studies classified brain injury as mild, moderate, or severe according to the anatomical site of signal alteration and as grey matter or cerebellar injury. Additionally, in the study by Niezen et al., the Van Rooij score [14] was used, while Arriaga-Redondo used the Scheme of Rutherford score [15] to classify brain injury. Both scales are validated to determine cerebral alterations in neonates with perinatal asphyxia in the first week of life.

In the articles by Niezen C. et al. (2018) and Arriaga-Redondo M. et al. (2019), rScO2 values >90% were defined as predictors of an abnormal brain MRI outcome [20] and were also associated with the combination of abnormal MRI and/or death (OR 1.7; 95% CI 1.29, 2.48, *p* = 0.001) [22]. Likewise, Peng S. et al. (2015), Lemmers P. et al. (2013), and Wintermark P. et al. (2014) found that rScO2 values are significantly higher in neonates who develop subsequent brain injury on brain MRI [23,24,25].

For their part, Jain S. et al. (2017) report that rScO2 had a more rapid increase in infants with more serious injuries observed on brain MRI, evidencing an average increase of 0.2% per hour for patients with no or mild injuries, 0.48% per hour in patients with moderate injuries, and 0.68% in severe injuries [26].

Regarding the anatomical location of the lesion, Szakmar E. et al. (2021) report that newborns with grey matter lesions have a significantly higher rScO2 on the second day of hypothermia and at the time of rewarming, which associates higher values with greater severity of brain injury, considering that grey matter lesions are classified as severe injuries in most studies [27].

In turn, Mitra S. et al. (2020) used aEEG to assess neurological outcomes concerning dynamic changes in brain metabolism, using the ratio [oxCCO] (oxidised cytochrome c Oxidase) and [HbD] (Hb difference (HbD = HbO2 - HHb)). They found a significant difference between groups with normal vs. abnormal aEEG, indicating mitochondrial injury and altered oxidative metabolism in the abnormal group [28].

### Long-term outcomes

Different validated scales were used to identify and define long-term neurological outcomes to diagnose neurodevelopmental disorders. The principal tool used in the studies was the BSID III scale (Bailey Scales of Infant Development version 3) between 18 and 36 months [11]. In addition, the GMDS scale was also frequently used from three months to five years (Griffith Mental Development Scales) [14], and one study evaluated neurodevelopment using the Capute and Mullen Scale [15].

Pereira C. et al. (2021) evidence that the comparison of rScO2 between the different neurodevelopmental groups revealed statistically significant differences at 48 hours of life between the moderately disabled and severely disabled groups with a rScO2 value of 83.5% (*p* = 0.019) and the severely disabled and normal neurodevelopmental groups (*p* = 0.013). Significant differences were also detected after hypothermia between the severely disabled and normal neurodevelopmental groups with a rScO2 value of 66% (*p* = 0.003) and between the moderately disabled and severely disabled groups (*p* = 0.043) [16].

In Jain S. et al. (2017), a higher rScO2 of the cut-off value (80%) beyond 24 hours correlated with higher odds of worse BSID III scores. Similarly, higher hourly mean absolute rScO2 correlated with higher odds of lower BSID scores in motor, cognitive, and language domains beyond 24 hours of life. However, increasing rScO2 did not show a significant relationship with the BSID scores [26]. Furthermore, for Burton V. et al. (2015), mean rScO2 in any period (hypothermia, rewarming, or normothermia) was not associated with a future deterioration in the Mullen score [30].

### Value of rScO2 by NIRs

Regarding the value of rScO2 by NIRS as a predictor of brain injury, there was significant discord among the different articles reviewed; however, a weighted average between 75 and 90% is taken as a predictor of brain injury in term neonates with moderate to severe hypoxic-ischaemic encephalopathy. Regarding the time of NIRS measurement, all studies performed data collection during hypothermia and after rewarming, i.e., on average, NIRS monitoring was between four and six days.

In the articles by Niezen C. et al. (2018) and Arriaga-Redondo M. et al. (2019), rScO2 values > 90% were defined as predictors of an abnormal brain MRI outcome [20] and were also associated with the combination of abnormal MRI and/or death (OR1.7; 95% CI 1.29, 2.48, *p* = 0.001) [15]. Likewise, Peng S. et al. (2015), Lemmers P. et al. (2013), and Wintermark P. et al. (2014) found that rScO2 values are significantly higher in neonates who develop subsequent brain injuries on brain MRI [17,18].

Peng S. et al. (2015), Jain S. et al. (2017), and Lemmers P. et al. (2013) reflect that high values in the first 36 hours are better predictors of injury; rScO2 greater than 75.5% in the first ten hours of hypothermia and rScO2 greater than 80% in the first 30 hours of life, respectively [17,19,20]. In contrast, Toet M. et al. (2006) state that neonates with adverse outcomes maintained stable rScO2 levels between 55% and 70%, which increased significantly after 24 hours [21]. On the other hand, Arriaga-Redondo M. et al. (2019), Oliveira Pereira C. et al. (2021), Niezen C. et al. (2018), and Szakmar E. et al. (2021) recognise the predictor value within the first 48 hours [14,15,16] with a rScO2 value greater than 84% during rewarming [22].

### Other indexes used

One study also evaluated the tissue oxygenation index (TOI) 6, 12, and 24 hours after birth. Ancora G. et al. (2013) describe TOI values at 12 hours, showing significantly higher values in newborns who had adverse events (n = 4) vs. those who did not have adverse outcomes (n = 8) (79.7 +/− 9.4% vs. 67.1+/− 7.9 p = 0.034) [23].

For three other studies, fractional tissue oxygen extraction (FTOE) was assessed. Goeral et al. (2017) describe that regional cerebral oxygen saturation increased over time with a parallel decrease in fractional cerebral tissue oxygen extraction (cFTOE) [24]. Similarly reported by Toet M. et al. (2006), in which from 24 hours onward, the FTOE values of infants with an adverse outcome were significantly lower compared with those with a favourable outcome (24 hours: P.05; 30 hours: P.001; 36 hours: P.001; 48 hours: P.001). Furthermore, the FTOE value in the adverse outcome group decreased significantly over time (*P* < 0.001, 48 vs. 12 hours) [21] and Lemmers P. et al. (2013), in which the mean cFTOE value became very low from 24 hours of age in the adverse outcome group [19].

### Systemic NIRS

It is striking that systemic NIRS is also associated with unfavourable neurological outcomes. In Shellhaas R. et al. (2013), Systemic rScO2 Variability was the best individual predictor of short-term outcome scores, while absolute values and rScO2 variability were independent of short-term outcomes on brain MRI and Thompson score [25].

## Discussion

The systematic review addressed the use of neuromonitoring of rScO2 by NIRS during hypothermia treatment in the prediction of and relationship to short- and long-term neurological injury in infants with perinatal asphyxia. The synthesis of this information shows that an increase in cerebral blood flow between 24 and 48 hours is related to an increase in the probability of short- and long-term neurological lesions. Likewise, although the risk of bias in the included observational studies is low, there is high heterogeneity in the information, especially in the definition of outcomes, methodologies, and measurement times.

The increase in cerebral blood flow, subrogated to the elevated value of rScO2 measurement, is explained from the point of view of pathophysiology. During neonatal asphyxia, changes at the cellular level occur at different stages of asphyxial injury. In general, the initial chain of events is composed of hypercapnia, hypoxemia, and acidosis that generate a loss in cerebral autoregulation and an increase in initial cerebral blood flow; simultaneously, cellular changes occur in the CNS such as necrosis, secondary reperfusion, and finally apoptosis [26].

After the initial insult (asphyxia), a primary energy failure is generated, accompanied by the consumption of high-energy phosphates and glucose, with failure of the Na/K ATPase pump and mitochondrial function, favouring oedema and early cell death (necrosis); this initial alteration generates different alterations in factors related to maintaining the self-regulation of brain metabolism, among which is an increase of hydrogen ions as a consequence of an anaerobic cycle of glucose consumption, which in the absence of oxygen enters the Cori cycle generating lactic acid and releasing hydrogen ions to the extracellular space. When these are captured by the cell, an exchange of ions of equal charge takes place, producing hyperkalaemia, leading to an increase in extracellular potassium that decreases the repetition of smooth muscle cell action potentials, thus keeping the cell hyperpolarised and generating vasodilatation. In addition, phosphorylation of ATP to ADP secondary to pump failure generates adenosine, which binds to P2Y receptors and generates equal vasodilation. Similarly, hypercapnia and increased carbon dioxide pressure play an important role in vasodilation [26].

All this results in a phase of hyperaemia and increased cerebral blood flow, a situation that explains the increase in the NIRS value found in the different studies correlating this pathophysiological characteristic. Subsequently, a second energetic failure occurs in which excitatory amino acids are released with increased oxidative stress due to calcium influx, which perpetuates the production of proteases and the release of free radicals, favouring the induction of pro-apoptotic factors that perpetuate late cell death [13].

Therefore, considering the factors involved in the pathophysiological process, their correlation with the initial elevation of rScO2 during treatment, and together with the measurement of clinical biomarkers such as severe acidosis, hypotension (accompanied by an immature vascular autoregulation system in the neonate), hypoxemia, hypercapnia, and electrolyte disturbances such as hyperkalaemia, these clinical variables and their correlation with cerebral blood flow (rScO2 by NIRS) and the state of cerebral autoregulation during the disease are important so that the clinician can perform interventions at the patient’s bedside to maintain these physiological variables in adequate ranges and protect cerebral blood flow in normal ranges. This would help to maintain an acid-base and hydro-electrolyte balance, understanding that these are influential elements in cell damage [26].

Similarly, it is important to understand the role of brain metabolism during neonatal asphyxia, and this is reflected in other NIRS measurement indices, such as the change in cytochrome c oxidase oxidation as an indicator of mitochondrial metabolism and ATP synthesis. Bale G. et al. (2019) report a significant relationship between decreased oxygenation and decreased cytochrome C oxidase oxidation in neonates with severe neurological injury, indicating the coexistence of a mismatch between oxygenation and metabolism at the cellular level in EHI with an unfavourable outcome [26]. This is explained in the chain of events during secondary energy failure, in which increased oxidative stress generates a permeability pore in the mitochondria, which, in addition, reaches a fully oxygen-dependent redox state, leading to the release of cytochrome C and, consequently, apoptosis-inducing factors [13]. Therefore, asphyxiated neonates that reach secondary energy failure and the apoptotic process will present worse neurological outcomes. Therein lies the importance of early interventions to avoid this stage, as well as the identification of the nature and severity of the insult, considering that this could be antepartum and, therefore, produce greater progress in the damage.

Likewise, it is essential to maintain optimal mean arterial pressure; small changes in arterial pressure will affect the cerebral blood flow autoregulation curve in scenarios of loss of cerebral autoregulation [26].

Authors such as Lee J. et al. (2017) confirmed this in their study [27], showing that NIRS values increased simultaneously with altered mean arterial pressure measurement. To improve neurological outcomes, both hypotension and hypertension states should be avoided in this group of patients. The clinician should constantly correlate the blood pressure monitoring data with the rScO2 value during treatment and make frequent adjustments to avoid damage to the neonate’s cerebral autoregulation [28].

Authors such as Marin T. et al. (2011) [28] have proposed the utility of NIRS in neonates for the continuous measurement of regional tissue oxygenation in different organs, mainly the brain, kidney, and mesentery, which reflects the perfusion status and allows physicians to directly monitor fluctuations in this perfusion in real-time. This statement is corroborated by the studies reviewed in this investigation (see Table 1) since they agreed on the usefulness of NIRS as a marker of cerebral blood flow (CBF), which indirectly gives us a measure of cerebral tissue perfusion.

Studies evaluate the association of brain NIRS with short-term and long-term outcomes. In the short term, in the investigations included in the systematic review, neurological injury is defined especially by brain MRI findings before discharge, usually within the first week of life. MRI is currently a widely recommended tool with standardised data to determine the pattern and severity of brain damage and the prognosis of the neonate with HIE [29,30]

The characterisation of findings related to neonatal asphyxia and its severity spectrum have been defined in some common injury patterns, such as deep grey matter lesions, posterior internal capsule lesions, cortical infarction, white matter hyperintensity in T2, and punctate lesions in white matter [31].

In most of the included studies, neuroimaging is performed after hypothermia, during the fourth or fifth day of life, and/or during the second week of life, as in the case of Wisnowski’s study [32]. However, it is important to highlight that the study by Liu W. et al. [33] reports that the specificity and sensitivity of MRI are better within two weeks of birth. Given the changing and late-onset pattern of MRI, the usefulness of NIRS measurement as an early predictor of brain injury is raised in the literature included in the systematic review. Thus, for example, the research by Szakmar E. et al. [22] shows that neonates with grey matter injuries in MRI correlate with higher rScO2 values on the second day of hypothermia, and in the study by Niezen C. et al. [14], the NIRS value of > 90% at 48 hours >90% is associated with severe brain injury by MRI. Thus, the measurement of NIRS before, during, and after therapeutic hypothermia can be proposed as an early predictive biomarker of neurological outcomes compared to MRI. This considers that according to the findings of this study, high NIRS values during the first 24 to 48 hours are related to unfavourable neurological outcomes, while brain MRI during the first 24 hours of life has limitations when it comes to fully identifying the extent of the lesion [34].

On the other hand, other authors also describe short-term outcomes using electroencephalograms (EEG). Continuous use of EEG in neonates with moderate to severe HIE helps to estimate the bioelectrical function of the brain in addition to detecting subclinical seizures [35]. In the study by Del Rio et al. [36], EEG can predict brain injury during the first 72 hours of life. In contrast, Niezen C. et al. [14] found that NIRS is a better predictor of neuronal injury at 72 hours than EEG. Since EEG can show abnormal electrical patterns in the brain, it is a useful tool at the bedside for early intervention, if necessary. However, the combination of bedside neuromonitoring tools within the neonatal unit, allowing a window into different physiological variables in the course of the disease, will be better than the isolated use of each in terms of neurological prediction and decision-making [8, 35].

Concerning long-term outcomes, the included studies assess neurodevelopment in the first 36 months of life using different instruments, all validated for this purpose. They specifically use the BSID III scales (Bailey Scales of Infant Development Version 3) between 18 and 36 months and the GMDS scale from three months to five years (Griffith Mental Development Scales). Regarding the BSID III scale, a cut-off point of < 85 has been described to define neurodevelopmental delay, and a close relationship has been found between low scores on the BSID III scale in cognitive, language, and motor domains in children with moderate hypoxic-ischaemic encephalopathy [37]. According to the data extracted in this systematic review, there is evidence of a relationship between high brain NIRS values and low scores in neurodevelopmental scales, which would make brain NIRS monitoring in the first hours of treatment and altered values useful for predicting neurodevelopmental alterations between 18 and 36 months. In their studies, Oliveira C. et al. demonstrated NIRS values at 48 hours above 83% correlate with a lower score in the BSID III and GMSD scales [16], and Toet et al. described a trend of increased cerebral rScO2 and decreased FTOE in the first 24 hours for neonates with neurodevelopmental disorders at two years of life. However, other studies have reported the limited ability of such scales to identify more subtle deficits in the cognitive spectrum, so children should be evaluated over the longer term, including school performance, to establish the impact of EHI on neurodevelopment across the lifespan [38]. This suggests that alterations in brain NIRS values may be related to neurodevelopmental delay, but normal NIRS values and scores on neurodevelopmental scales do not rule out the coexistence of some degree of compromise. Given the limited information found in other studies about NIRS values and long-term neurodevelopment, further research on this topic is suggested.

Among the strengths of this systematic review are the quality of the studies and the low risk of bias. The data obtained are reliable and extrapolatable, which generates good inferences from these data with a low risk of bias. However, one limitation is that most of the studies did not propose a sample size for the proposed outcome. Another limitation was the heterogeneity in different variables that would allow standardisation of the data and results. In this sense, we found significant variability between studies in the value of NIRS as a predictor of acute brain injury, the time during treatment that can be a predictor of the value obtained, and methodological aspects of the measurement such as the site of placement for the measurement and the instrument used. Therefore, working on the standardisation of the measurement is recommended to improve the interpretation of the result and its impact on patient care. In addition, heterogeneity was also found in the definition of short- and long-term outcomes. As brain MRI and neurodevelopmental scoring scales are not standardised across studies, it is necessary to define a standardised method for measuring brain injury in MRI as well as a scale for measuring neurodevelopment in asphyxiated neonates.

## Conclusion

A relationship is observed between high values of rScO2 in cerebral NIRS during hypothermia treatment, especially in the first hours of treatment, and the prediction of short-term (brain MRI) and long-term (neurodevelopmental alterations) neurological injury. Neuromonitoring with NIRS as a biomarker is a useful bedside tool to predict brain injury in neonates with moderate to severe HIE. It also provides real-time information for timely and early follow-up and therapeutic interventions in the neonatal care unit.
